# Impact of dental caries on the daily lives of geriatric patients visiting dental hospitals in Rawalpindi, Pakistan

**DOI:** 10.34172/joddd.40741

**Published:** 2024-03-29

**Authors:** Saadia Ayub, Saman Waqar, Muhammad Tahir Muneeb

**Affiliations:** ^1^Al-Shifa School of Public Health, Al-Shifa Trust Eye Hospital, Rawalpindi, Pakistan; ^2^Department of Anesthesiology, Fazaia Medical College, Pakistan Airforce Hospital, Islamabad, Pakistan

**Keywords:** Daily living, Dental health, DMFT, Geriatric population, Oral health-related quality of life

## Abstract

**Background.:**

The objectives were to assess the impact of dental caries on the daily living of the geriatric population and determine the factors that influence the relationship between dental health and the daily living of the geriatric population.

**Methods.:**

A descriptive cross-sectional study was carried out over six months at Rawalpindi’s public and private dental hospitals. Participants aged≥60 years, both male and female, were selected. The calculated sample size was 281. Desired sample from one of the dental hospitals was collected using a non-probability consecutive sampling strategy. Data about sociodemographic characteristics and the DMFT index were collected. Adapted validated tool dental impact on daily living (DIDL) was used to assess the impact of dental health on daily living.

**Results.:**

Chi-squared test of association showed a positive association between the DIDL and sociodemographic variables, including age (*P*=0.001), gender (*P*=0.001), education (*P*=0.001), income (*P*=0.001), occupation (*P*=0.029), marital status (*P*=0.001), living arrangement (*P*=0.001), and history of chronic illnesses (*P*=0.001). The association between the DMFT index and DIDL also showed statistically significant results (*P*=0.001). Binary logistic regression analysis indicated that gender (OR=6.98, *P*=0.005) and the individual’s dental health (OR=6.43, *P*=0.001) were the strongest predictors of the impact experienced in daily life activities. The overall model was statistically significant (χ^2^=51.24, *P*=0.001), and the variables were responsible for 32.4% of the variance in the outcome variable.

**Conclusion.:**

The study provides strong evidence that sociodemographic factors, DMFT index, gender, and individual dental health significantly contribute to the impact of dental health on daily living. Gender and individual dental health emerge as particularly influential predictors. These findings emphasize the need for targeted interventions and awareness programs, especially for groups with a higher risk of experiencing a significant impact on daily life due to dental issues.

## Introduction

 The World Health Organization (WHO) stated in 1948 that health is “complete physical, mental, and social well-being and not merely the absence of disease or infirmity.” Despite the definition of health by the WHO, universal health has routinely been considered a unidimensional concept with a focus on disease identification and treatment. Oral health refers to the overall health of the mouth, teeth, gums, and entire orofacial system.^1,2^ According to the World Dental Federation, Oral health is multidimensional and includes not only the health of teeth and gums but also the ability to speak, smile, smell, taste, swallow, and show emotions through facial expressions without any pain, discomfort, or medical condition related to craniofacial complex.^3^ It is part of an individual’s general health and well-being, with dental caries and periodontitis being the most prevalent conditions affecting dental health worldwide.^4^ Unfortunately, oral health is not explicitly targeted in the development agenda for Sustainable Developmental Goal SDG3. According to a popular Chinese saying, “The illness comes from the mouth,” emphasizing the critical role of dental health in the overall well-being of an individual. Therefore, the authors of a study suggested that oral health should be considered as the initial step toward achieving SDG3. Thus, oral health is not just limited to dental epidemiology focused on teeth. It has a significant impact on systemic health conditions. The interplay between oral health, overall health, and well-being cannot be disregarded. It is crucial to raise global awareness about oral health care, promotion, and universal coverage in dentistry and related oral sciences.

 With a change of the medical model to a social model in the health care system, the traditional biomedical endpoints of clinical studies have been extended to include patient-centered measurements, such as quality of life. Dental health is part of general health and is recognized as a vital component of quality of life.^5^ Oral health-related quality of life (OHRQoL) is a multidimensional concept that includes a subjective assessment of oral health, functional well-being, emotional well-being, expectations, and satisfaction with care and self-worth.^6^ The dental impact on daily living (DIDL) scale is a promising measure to evaluate OHRQoL. This socio-dental scale encompasses five dimensions that assess various aspects of an individual’s well-being. The objective approach focuses on measurable external life conditions like family income, educational level, and access to healthcare facilities. The subjective approach focuses on the patient’s self-evaluation of life conditions, like the extent of the problem, satisfaction, happiness, and sorrow. Despite its relatively recent emergence in recent decades, OHRQoL has significant implications for the clinical practice of dentistry and dental research. The goal of health is to provide complete coverage of health-related subjects and analyze patterns in factors that impact health such as healthcare use, healthcare resources, health expenses and healthcare providers. Additionally, it aims to explore the determinants of health and assess changes in health status over time (National Center for Health Statistics, US, 2023).

## Methods

 This descriptive cross-sectional study was carried out in six months, from April to September 2023, at public and private dental hospitals of Rawalpindi and Islamabad. Margalla Institute of Health Sciences Rawalpindi was selected using simple random sampling. Patients visiting the outpatient unit were selected using a consecutive sampling technique based on inclusion and exclusion criteria. All male and female participants aged ≥ 60 visiting the outpatient department of the dental hospital were included in the study. Participants who did not consent to participate, were sick during the study time, and used fixed prostheses for tooth loss were excluded. The sample size was calculated using the proportion formula for sample size calculation in OpenEpi Menu version 3.01. The previous prevalence of oral health and its effect on life quality was 76%.^7^ The calculated sample size was 281 with a 95% confidence interval (CI), 5% margin of error, and 80% study power. Informed consent was taken verbally from all the patients before data collection.

 Data was collected using an interview-based questionnaire in which the researcher asked the questions from respondents and recorded their responses accordingly. The questionnaire was devised after reviewing different research papers. A proforma was developed first in English, then later translated into Urdu, the national language of Pakistan, to collect data regarding sociodemographic characteristics of the respondents, DMFT (decayed, missing, filled teeth) index and DIDL, among geriatric population visiting the outpatient department of the dental hospital. The validity of the questionnaire was checked through public health specialists and healthcare providers. Reliability analysis was performed, and Cronbach’s alpha coefficient was calculated at 0.883. A pilot study was carried out, structured questionnaires were filled out by 10% of the total sample size (28 participants), and the tool was modified after pilot testing accordingly. After finalizing the research questionnaire, data were collected.

 The first section of the questionnaire included questions about sociodemographic characteristics; the second part included the DMFT index, i.e., the number of decayed, missing, and filled teeth, which is used to measure the prevalence of dental caries and tooth decay in a population. The questions are designed to be completed by dental professionals during dental examinations. The DMFT score is obtained by adding the number of decayed, missing, and filled teeth for each individual. The score can range from 0 to 32. A higher DMFT score indicates the individual’s poorer oral health status.

 The third part included the DIDL tool developed by Leao and Sheiham,^8^ which measures the extent to which dental problems interfere with an individual’s ability to perform daily activities such as eating, speaking, and socializing. The scoring system uses a 4-point Likert scale, which assigns a value to each response ranging from 0 (no impact on daily living) to 3 (severe impact). Options for questions are “never,” “rarely,” “sometimes,” and “often.” These responses are assigned values of 0, 1, 2, and 3, respectively. After completing the questionnaire, which comprises a total of 23 questions, the scores of each question are summed up to obtain a total DIDL score, which can range from 0 to 69. Higher scores indicate a greater impact of dental problems on daily living.

 A codebook was developed, and data was registered in SPSS 26. The outcome variable was the DIDL. Data was transformed, and the DIDL was divided into categories using SPSS. Data were divided into three categories. The first category was low impact on daily living, ranging from 0 to 14, with moderate impact on daily living ranging from 15 to 29, and high impact on daily living ranging from 30 to 44. The chi-squared test of independence was applied to examine the association between sociodemographic variables and the DMFT index. The chi-squared test was also applied to DIDL with each sociodemographic variable. Furthermore, binary logistic regression analysis was applied to get a more detailed insight into the predictors of dental impact on the daily lives of the geriatric population.

## Results

###  Demographic characteristics

 A total of 281 respondents were included in this study. The minimum reported age was 60, and the maximum age was 77 years. The mean age of the respondents was 64.39 ± 4.43. Among those, 154 (54.54%) were male, and 127 (45.2%) were female; 74 (26.3%) respondents were illiterate, 87 (31%) had an educational level up to matric, and 70 (24.9%) were graduates. Government sector employees comprising 50 (17.8%) and 125 (44.5%) individuals were doing private sector jobs, and 106 (37.7%) were unemployed. Most respondents (n = 92, 32.7%) had an income of less than 20 000, and 66 (23.5%) had a revenue of more than 100 000. The number of respondents who reported as non-smokers was 231 (82.2%), and none reported the intake of paan and betelnut. Among the smokers (n = 50, 17.8%), the majority (n = 19, 6.8%) reported four cigarettes daily. History of chronic illness showed that 66 (23.5%) respondents had diabetes, 48 (17.1%) had heart diseases, and 53 (18.9%) had multiple diseases at the time of this study. Table 1 presents the demographic characteristics of the participants.

**Table 1:** Demographic characteristics of the participants

**Sociodemographic variables**	**Categories**	**Frequency**	**Percent**
Gender	Male	154	54.58
	Female	127	45.2
Education	Illiterate	74	26.3
	Matric	87	31.0
	Intermediate	35	12.5
	Graduate	70	24.9
	18 Years or more	15	5.3
Occupation	Government sector	50	17.8
	Private sector	125	44.5
	Unemployed	106	37.7
Income	Less than 20 000	92	32.7
	20 000-50 000	50	17.8
	50 000-100 000	73	26.0
	More than 100 000	66	23.5
Marital status	Unmarried	4	1.4
	Married	234	83.3
	Separate/divorced	5	1.8
	Widow	38	13.5
Living arrangement	Alone	10	3.6
	With spouse	99	35.2
	With children	70	24.9
	Spouse & children	102	36.3

###  Descriptive results for DMFT index

**Figure 1:**
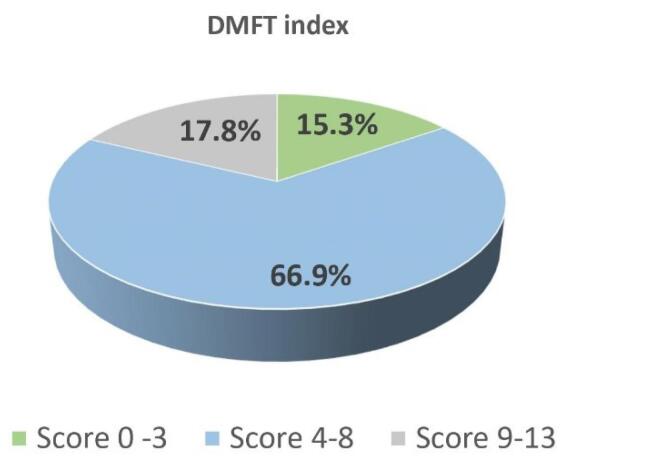
The descriptive results of DMFT categories

 The respondents reported a maximum of 6 decayed teeth, with a mean of 2.12 ± 1.43, with a maximum of 8 missing teeth, with a mean of 2.73 ± 1.92, and a maximum of 5 missing teeth, with a mean of 1.23 ± 1.21. The DMFT index was calculated by counting the number of teeth in each category and adding up the scores. Generally, the DMFT score range is 0‒32. A minimum of 0 DMFT score and a maximum of 12 were reported. The mean DMFT score was 6.12 ± 2.60. It was divided into categories using SPSS software. The first category ranged from 0 to 3, the second ranged from 4 to 8, and the third ranged from 9 to 13. A higher DMFT score indicates the individual’s poorer oral health status. Figure 1 shows the descriptive results of DMFT categories using a pie chart.

**Figure 2:**
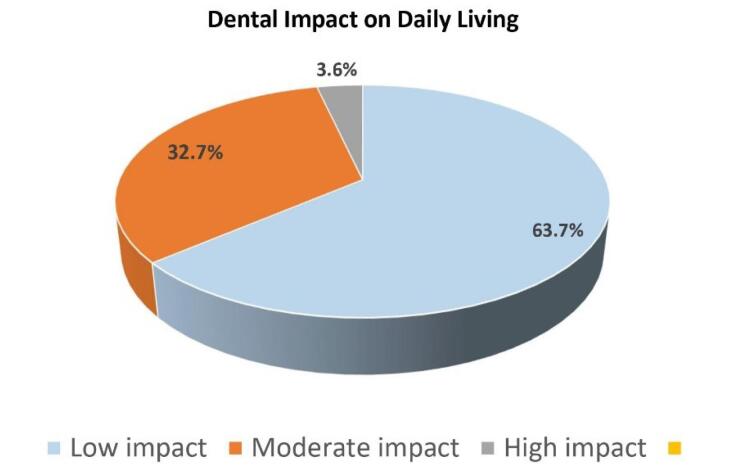
Dental impact on daily living

 The scoring was accomplished using a 4-point Likert scale, which assigns a value to each response ranging from 0 (no impact on daily living) to 3 (severe impact). The options for the questions were “never,” “rarely,” “sometimes,” and “often,” with scores of 0, 1, 2, and 3, respectively. A total of 23 questions were included in this study, and the scores of each question were summed up to obtain a total DIDL score with a range of 0‒69. A minimum of 0 and a maximum of 44 scores were obtained with a mean of 12.85 ± 8.28 (Table 2). Higher scores indicate a greater impact of dental problems on daily living. Data were transformed, and the DIDL was divided into categories using SPSS. Data were divided into three categories. The first category was low impact on daily living ranging from 0 to 14; moderate impact on daily living ranged from 15 to 29; and high impact on daily living ranged from 30 to 44, as shown in Figure 2.

**Table 2:** Frequency of answers to dental impact on daily living (DIDL) tool

		**Frequency**	**Percent**
Has your dental condition caused you pain or discomfort in the last month?	Never	21	7.5
	Rarely	81	28.8
	Sometimes	118	42.0
	Often	61	21.7
Has your dental condition caused you difficulty chewing in the last month?	Never	23	8.2
	Rarely	51	18.1
	Sometimes	75	26.7
	Often	132	47.0
Has your dental condition caused you difficulty in swallowing food in the past month?	Never	226	80.4
	Rarely	48	17.1
	Sometimes	7	2.5
	Often	0	0
Has your dental condition caused you difficulty in speaking in the past month?	Never	264	94.0
	Rarely	15	5.3
	Sometimes	2	0.7
	Often	0	0
Has your dental condition caused you difficulty in opening mouth in the past month?	Never	275	97.9
	Rarely	4	1.4
	Sometimes	2	0.7
	Often	0	0
Has your dental condition caused you difficulty in reading in the last month?	Never	268	95.4
	Rarely	8	2.8
	Sometimes	5	1.8
	Often	0	0
Has your dental condition affected your sense of taste in the last month?	Never	266	94.7
	Rarely	15	5.3
	Sometimes	0	0
	Often	0	0
Has your dental condition affected your appetite in the last month?	Never	201	71.5
	Rarely	53	18.9
	Sometimes	23	8.2
	Often	4	1.4
Has your dental condition caused you difficulty in cleaning your teeth in the past month?	Never	185	65.8
	Rarely	63	22.4
	Sometimes	31	11.0
	Often	2	0.7
Has your dental condition caused you difficulty in maintaining personal hygiene	Never	245	87.2
	Rarely	27	9.6
	Sometimes	9	3.2
	Often	0	0
Has your dental condition affected your socioeconomic status in the past month?	Never	104	37.0
	Rarely	54	19.2
	Sometimes	84	29.9
	Often	39	13.9
Has your dental condition caused you trouble in performing religious activities?	Never	142	50.5
	Rarely	99	35.2
	Sometimes	31	11.0
	Often	9	3.2
Has your dental condition caused you difficulty in smiling in the past month?	Never	199	70.8
	Rarely	70	24.9
	Sometimes	2	0.7
	Often	10	3.6
Has your dental condition caused you difficulty in laughing in the past month?	Never	224	79.7
	Rarely	43	15.3
	Sometimes	4	1.4
	Often	10	3.6
Has your dental condition caused you embarrassment in the past month?	Never	189	67.3
	Rarely	78	27.8
	Sometimes	10	3.6
	Often	4	1.4
Has your dental condition caused you difficulty in socializing in the past month?	Never	209	74.4
	Rarely	58	20.6
	Sometimes	10	3.6
	Often	4	1.4
Has your dental condition affected your relationship with family in the past month?	Never	237	84.3
	Rarely	40	14.2
	Sometimes	4	1.4
	Often	0	0
Has your dental condition affected your relationship with friends in the past month?	Never	259	92.2
	Rarely	18	6.4
	Sometimes	4	1.4
	Often	0	0
Has your dental condition affected your relationship with colleagues in the past month?	Never	259	92.2
	Rarely	22	7.8
	Sometimes	0	0
	Often	0	0
Has your dental condition caused you difficulty in work in the past month?	Never	145	51.6
	Rarely	80	28.5
	Sometimes	33	11.7
	Often	23	8.2
Has your dental condition caused you little interest or pleasure in doing things?	Never	106	37.7
	Rarely	99	35.2
	Sometimes	51	18.1
	Often	25	8.9
Has your dental condition made you less motivated to do work?	Never	88	31.3
	Rarely	112	39.9
	Sometimes	56	19.9
	Often	25	8.9
Has your dental problem made it difficult for you to perform daily life activities?	Never	89	31.7
	Rarely	103	36.7
	Sometimes	62	22.1
	Often	27	9.6

###  Inferential results

 The chi-squared test was applied to DIDL scores with each sociodemographic variable. The association between the DMFT index and the DIDL index was also recorded. Table 3 shows the results for each variable.

**Table 3:** The association between each variable and the DIDL index

**Sociodemographic factors**	**Dental impact on daily living score (DIDL score)**				
	**0-14** **(Low impact)**	**15-29** **(Moderate impact)**	**30-44** **(High impact)**	***χ*^2^ (*df*)**	***P *** **value**
Age groups (y)					
60-65	117 (62.6%)	64 (34.3%)	6 (3.2%)	20.7 (4)	0.0001
66-71	34 (51.5%)	28 (30.4%)	4 (6.1%)		
72-77	28 (15.6%)	0 (0.0%)	0 (0.0%)		
Gender					
Male	119 (77.3%)	33 (21.4%)	2 (1.3%)	28.0 (2)	0.0001
Female	60 (47.2%)	59 (46.5%)	8 (6.3%)		
Education					
Illiterate	26 (35.1%)	40 (54.1%)	8 (10.8%)	78.0 (8)	0.0001
Matric	48 (55.2%)	39 (44.8%)	0 (0.0%)		
Intermediate	24 (68.6%)	11 (31.4%)	0 (0.0%)		
Graduate	66 (94.3%)	2 (2.9%)	2 (2.9%)		
18 years or more	15 (100%)	0 (0.0%)	0 (0.0%)		
Income					
Less than 20 000	52 (56.5%)	36 (39.1%)	4 (4.3%)	35.8 (6)	0.0001
20 000-50 000	40 (80.0%)	10 (20.0%)	0 (0.0%)		
50 000-100 000	31 (42.5%)	38 (52.1%)	4 (5.5%)		
More than100 000	56 (84.8%)	8 (12.1%)	2 (3.0%)		
Occupation					
Government	35 (70.0%)	13 (26.0%)	2 (4.0%)	10.8 (4)	0.029
Private	89 (71.2%)	32 (25.6%)	4 (3.2%)		
Unemployed	55 (51.9%)	47 (44.3%)	4 (3.8%)		
Marital status					
Unmarried	0 (0.0%)	4 (100%)	0 (0.0%)	25.0 (6)	0.0001
Married	158 (67.5%)	67 (28.6%)	9 (3.8%)		
Divorced/separated	4 (80.0%)	0 (0.0%)	1 (20.0%)		
Widow	17 (44.7%)	21 (55.3%)	0 (0.0%)		
Living arrangement					
Alone	2 (20.0%)	8 (80.0%)	0 (0.0%)	31.5 (6)	0.0001
With spouse	79 (79.8%)	16 (16.2%)	4 (4.0%)		
With children	47 (67.1%)	21 (30.0%)	2 (2.9%)		
Spouse & children	51 (50.0%)	47 (46.1%)	4 (3.9%)		
History of tobacco use					
Smokers	34 (68.0%)	14 (28.0%)	2 (4.0%)	0.62 (2)	0.731
Non-smokers	145 (62.8%)	78 (33.8%)	8 (3.5%)		
History of chronic illness					
Diabetes	38 (57.6%)	26 (39.4%)	2 (3.0%)	50.7 (8)	0.0001
Heart disease	16 (33.3%)	28 (58.3%)	4 (8.3%)		
Others (respiratory disease, cancer)	18 (100%)	0 (0.0%)	0 (0.0%)		
Multiple diseases	49 (92.5%)	4 (7.5%)	0 (0.0%)		
None	58 (60.4%)	34 (35.4%)	4 (4.2%)		

 The results showed a significant association between the DIDL index and Age (*P* = 0.0001). Respondents in the 60‒65 age group had the least impact on their daily lives due to dental health (62.6%). Participants in the 72‒77 age group reported to the dental hospital in lower numbers. They reported the least impact on their lives due to their dental health, as most of them were using prostheses to replace their lost teeth and had other issues in their daily lives that were given priority over dental health.

 A positive association was reported between the DIDL and gender (*P* = 0.0001). Male participants reported a low impact on daily lives (n = 119). In contrast, females reported a low-to-moderate impact on daily lives (n = 219), and (n = 8, 6.3%) reported a high impact on daily lives due to dental health, as shown in Table 3.

 A positive association was reported between the DIDL) and education (*P* = 0.0001). The higher the education, the lesser impact dental health had on the daily living of the geriatric population, i.e., 94.3% of respondents were graduates and had a low impact of dental health on daily living; 100% of the respondents with 18 years of education had low impact of dental health on daily living. Similarly, DIDL had a statistically significant association with income (*P* = 0.0001). Respondents with an income of more than 100 000 reported a low impact of dental health on daily living. Also, a statistically significant association was found between the DIDL and occupation (*P* = 0.029). The majority of respondents were private employees with a lower impact of dental health on daily lives, which could be due to the convenience and affordability of dental services.

 The association between the DIDL and marital status (*P* = 0.0001) was also statistically significant. Married individuals reporting to dental hospitals reported a low impact of dental health on daily living, which could be due to emotional support provided by the spouse, making it easier to cope with dental issues or seek necessary dental care. Similar results were obtained with the living arrangement (*P* = 0.0001), as shown in Table 3.

 The association of the DIDL with a history of chronic illness (*P* = 0.0001) was also statistically significant. Individuals with multiple diseases reported a low impact on their daily lives due to dental health. This could be because dental health was not a priority for those already having numerous life issues. Individuals with no disease reported a low-to-moderate impact on daily lives due to dental health, as only dental health affected their functional abilities.

 In this study, the association between the DMFT index and DIDL was significant (*P* = 0.0001).Good and moderate dental health was reported by the individuals who had a low impact on daily living. The individuals with poor dental health reported a moderate-to-high impact of dental health on daily living, as shown in Table 4.

**Table 4:** The association between the DMFT index and DIDL

**DMFT index**	**Dental impact on daily living score (DIDL score)**			***χ*^2^ *(df)***	***P *** **value**
	**0-14** **(Low impact)**	**15-29** **(Moderate impact)**	**30-44** **(High impact)**		
0-3 (Good dental health)	39(90.7%)	4(9.3%)	0(0.0%)	76.8 (4)	0.0001
4-8 (Moderate dental health)	127(67.6%)	61(32.4%)	0(0.0%)		
9-13 (Poor dental health)	13(26.0%)	27(54.0%)	10(20.0%)		

 The chi-square test of association was followed by binary logistic regression analysis using SPSS to understand the relationship between one or more predictor variables and the binary outcome variable. For this purpose, the categories of significant variables were converted into dichotomous variables. The independent variables were also converted into dichotomous variables. The main goal of this analysis was to model the probability of occurrence of one of the two categories based on the predictor variable, as shown in Table 5.

**Table 5:** The probability of occurrence of one of the two categories based on the predictor variable

**Predictor variables**	**B (SE)**	***P*** ** value**	**O R**	**95% CI (LL, UL)**
Gender Male = 0 Female = 1	1.94 (0.69)	0.005	6.98	1.7,27.2
Education (y) Less than 12 = 0 More than 12 = 1	0.42 (0.66)	0.522	1.52	0.4,5.6
Occupation Employed = 0 Unemployed = 1	0.90 (0.55)	0.102	2.46	0.8,7.2
Living arrangement Alone = 0 Family = 1	-3.07 (1.05)	0.004	0.04	0.0,0.3
Marital status Single = 0 Married = 1	0.21 (0.56)	0.706	1.23	0.4,3.7
DMFT (Dental health) Score 0-21(good) = 0 Score 22-44(poor) = 1	1.86 (0.49)	0.0001	6.43	2.4,16.9

LL: lower limit, UL: Upper limit.

 The model’s overall significance was tested using the Wald chi-squared statistics. The result was significant (χ^2^ = 51.24, *P* = 0.0001), indicating a significant improvement in fit with predictor variables compared to the null model; hence, it proved a good fit model. The Hosmer-Lemeshow test reported a significant value of 0.097, which is greater than 0.05, indicating a good fit, with no significant difference in the observed and predicted model. The Nagelkerke R square value of (0.324) indicated that approximately 32.4% of the variance in the outcome variable could be explained by the predictor variables included in the model.

 The results of logistic regression analysis indicated a significant association between gender and the level of impact experienced in daily life activities (*P* = 0.005). The odds of females (category 1) were approximately 6.98 times higher for moving to a higher category of the outcome variable, which is the impact on daily living compared to male individuals (reference category 0), while keeping all other variables constant. The other significant predictor variable is the dental health of the individual obtained using decayed, missing, and filled teeth. The relationship between dental health and the impact on daily living was statistically significant (*P* < 0.001). With each unit increase in the DMFT score, the odds of moving to a higher category of the outcome variable increased by 6.43 times.

 The living arrangement was negatively associated with an increased impact on daily living (*P* = 0.004). Living with family significantly decreased the odds of the impact on daily living. Education (*P* = 0.52), occupation (*P* = 0.102), and marital status (*P* = 0.706) showed a statistically insignificant impact on daily living. The odds ratio, however, suggested that a one-unit increase in education increased 1.52 times the odds of falling into the higher impact category on daily living; unemployed individuals had 2.46 times more odds of falling into the higher impact on daily living. While the odds ratio implies a potential effect, the high *P* value suggests this could be due to change. Further investigation and replication of this finding are required to draw firm conclusions.

## Discussion

 This cross-sectional study assessed the impact of dental health on the daily living of the geriatric population visiting dental hospitals in Rawalpindi. The association between the DIDL and sociodemographic variables showed a statistically significant association between DIDL index and age (*P* = 0.0001), gender (*P* = 0.0001), education (*P* = 0.0001), income (*P* = analysis indicated that gender [OR = 6.98, *P* = 0.005] and the individual’s dental health obtained using the DMFT index [OR = 6.43, *P* = 0.0001] are the strongest predictors of the impact experienced in daily life activities.

 In the present study, respondents with an income of more than 100 000 (n = 56,84.8%) reported a low impact of dental health on daily living, which could be due to stable incomes; they found it more convenient to afford dental care than those with unstable or lower incomes. Also, a statistically significant association was found between the DIDL and occupation (*P* = 0.029). Maximum respondents were private employees with a lower impact of dental health on daily lives due to the affordability of dental services, flexible working hours to visit the dentist, accessibility to dental hospitals, and employee benefits, including health insurance that might cover dental care. A study was conducted in Iran to assess the impact of oral health on the daily activities of individuals. The study found that 82.6% of the participants had experienced one or more oral impacts on their daily activities. These impacts could be due to various oral health issues affecting their ability to perform daily tasks comfortably. Nearly half of the impacts reported (49.5%) were considered severe or very severe, indicating that oral health problems substantially and negatively affected participants’ ability to carry out their daily activities. The oral impact on daily performance was higher in participants with a lower wealth index, suggesting that individuals with lower socioeconomic status were more significantly affected by oral health issues. ^9^ It was suggested in another study that among subjects from lower social classes, there appeared to be a weak correlation between DIDL and their oral health status because they prioritized different aspects of life rather than dental health.^10^

 A study conducted in 2019 evaluated the oral health status of the population based on the DMFT index. The results showed that DMFT was significantly associated with age (*P* = 0.001), marital status (*P* = 0.000), education (*P* = 0.001), and socioeconomic status (*P* = 0.001). Poor DMFT scores were achieved in people 35‒45 years of age (DMFT = 7.83), widows (DMFT = 9.05), people with low literacy rate (DMFT = 8.1), and people belonging to low socioeconomic class (DMFT = 8.9). The study revealed a notable trend as individuals grew older: the DMFT index tended to become increasingly unfavorable.^11^ This can be attributed to a higher prevalence of decayed, missing, and filled teeth with advancing age. Consequently, the WHO established a higher DMFT Index benchmark for the older age group. These results were consistent with the present study.

 A study was conducted in 2020 to assess the association between caries and OHRQoL to understand its impact on people’s daily living. The results showed that 87.6% of the people visiting the hospital had one or more decayed teeth. Among various dimensions assessed by the DIDL scale, two of the most frequently reported impacts on daily living were difficulty eating and disturbances in relaxation and sleeping patterns. In the present study, the most frequently reported impact on daily living was the ability to chew. A total of 132 (47.0%) of the individuals reported difficulty in chewing by choosing the option “often.” Adults with caries demonstrated increased odds of reporting higher oral impacts than those without caries. The presence of caries in individuals was associated with a higher likelihood of reporting more frequent and severe oral impacts that had tangible effects on their daily lives. As the exposure to caries increased, there was a linear rise in the oral impact on daily performance score, signifying a direct correlation between caries and the negative impact on OHRQoL.^12^

 A study was conducted in 2022 in Kosovo to evaluate the impact of dental status on daily living and OHRQoL. The results showed that while comparing the independent variables, gender was significantly different when total scores of DIDL were compared. Female patients reported higher satisfaction with their quality of life than male patients. Linear regression analysis also confirmed the impact of gender on DIDL scores.^13^ Some other studies also confirmed that females are more concerned about their dental and physical appearance than males.^14^ Another study also reported that females act more positively towards dental health than males.^15^ These findings are in contrast to our study in which male participants reported lower impact on daily lives (n = 119) while females reported lower to moderate impact on daily lives (n = 219) and (n = 8, 6.3%) reported high impact on daily lives due to dental health. The odds of females are approximately 6.98 times higher for transitioning to high impact on daily living compared to males. A study conducted in Turkey in 2008 reported a mean DMFT index of 11.4 and a significant caries index of 14.00, increasing with aging. Logistic regression analysis reported that older age and females were significant risk factors for high caries index.^16^

 The present study reported an association between DIDL and marital status (*P =*0.000). It also showed statistically significant results. Married individuals reporting to dental hospitals reported a low impact of dental health on daily living, which could be due to emotional support provided by the spouse, making it easier to cope with dental issues or seek necessary dental care. Similar results were obtained with the living arrangement (*P =*0.000). Another study in India reported that social, economic, and psychological factors significantly affected females’ dental health.^17^ Similarly, a study conducted in Korea in 2014 assessed the quality of life of single, married, separated, and divorced females and reported that married women had better scores. The highest quality of life scores were reported among married men aged 40‒69. Single women aged 40‒69 had lower scores than married women. Thus, it was concluded that there was a significant positive association between marital status and the quality of life in older ages.^18^

## Conclusion

 This cross-sectional study highlighted the effect of age, gender, income, education, marital status, and dental health conditions on the daily lives of the geriatric population. The findings of this study highlight the importance of addressing dental health issues and providing adequate dental care, especially in vulnerable populations. Improving access to dental services at affordable cost and promoting preventive oral health measures help reduce the negative impact of oral health problems on daily living and the overall well-being of an individual. Dental health should be prioritized by individuals of all ages, genders, social classes, marital relations, and educational levels. Regular dental checkups and treatments should be scheduled to maintain good oral health to positively impact overall well-being. Good oral hygiene practices and preventive care are essential for everyone as they can impact the daily living of individuals.

## Strengths

- The DIDL questionnaire allows for a more comprehensive assessment of the impact of dental health on various aspects of daily life, providing a holistic understanding of the participant’s experience.
- Each individual’s data were collected through interview-based questions.
- The study was conducted in a teaching dental hospital where individuals from all social classes visit for their dental checkups, especially those from lower social classes, as the treatment is affordable, thus providing valuable insights into oral health challenges faced by marginalized communities.

## Limitations

- The findings may not represent the broader population as the study was conducted in one of the dental hospitals, so it may be challenging to generalize the results to individuals from different social, economic, and educational backgrounds.

## Recommendations

- This study emphasizes the critical role of dental health in the overall well-being of an individual. The DIDL scale is a promising measure to evaluate OHRQoL.The oral health of the geriatric population is often associated with functional limitations, which can impact their ability to perform daily activities. The DIDL scale allows for a more comprehensive assessment of dental health’s impact on various aspects of daily life, providing a holistic understanding of the participant’s experience. It is therefore important to study this complex and multi-faceted issue with far-reaching implications for the overall health and well-being of an individual and address and recognize these issues to improve the daily living of older adults.

## Competing Interests

 None.

## Ethical Approval

 Permission was granted by the Institutional Review Board of Al-Shifa School of Public Health, Rawalpindi before the study was conducted.

## Funding

 This research project was not funded by any external source.
